# Refinement of Locomotor Activity during Development Is Correlated to Increased Dopaminergic Signaling in Larval Zebrafish

**DOI:** 10.1523/ENEURO.0444-25.2026

**Published:** 2026-05-05

**Authors:** Briee Mercier, Sandra M. Garraway, Matthew L. Beckman, Mark A. Masino

**Affiliations:** ^1^Departments of Family Medicine and Community Health, University of Minnesota, Twin-Cities, Minneapolis, Minnesota 55455; ^2^Neuroscience, University of Minnesota, Twin-Cities, Minneapolis, Minnesota 55455; ^3^Department of Cell Biology, Emory University, Atlanta, Georgia 30322; ^4^Department of Biology, Augsburg University, Minneapolis, Minnesota 55454

## Abstract

The refinement of gross motor skills, such as locomotion, during development is conserved across vertebrate species. Our previous work demonstrated, in larval zebrafish, that dopaminergic signaling through the dopamine D2-like family of receptors, specifically the dopamine 4 receptor subtype, was necessary for the developmental transformation of behaviorally relevant locomotor activity from an immature to a mature pattern between 3 and 4 d postfertilization. In this study, we used a complement of tools, including electrophysiology, pharmacology, in vivo calcium imaging, liquid chromatography-mass spectrometry, and quantitative reverse transcription polymerase chain reaction to characterize the functional and molecular mechanisms responsible for this dopaminergic-mediated refinement of spinal locomotor activity. The results demonstrate that the dopamine 4 receptor subtype is functional in, at least, a subset of immature larvae. Further, gene expression of all D2-like receptor subtypes, levels of dopamine, and activity of diencephalic dopaminergic neurons are significantly greater in mature larvae compared with immature larvae. The integration of these results provides correlative evidence for the developmental role of dopaminergic signaling, specifically the dopamine receptor 4 subtype, in the refinement of locomotor activity in vertebrates.

## Significance Statement

Throughout life, all vertebrates acquire and improve gross motor skills. This is particularly evident in the locomotor system where motor output is initially coarse and becomes progressively more refined during development. Previously, we demonstrated that dopaminergic signaling was a factor in the developmental refinement of locomotor activity. However, an understanding of the molecular and functional mechanisms underlying the dopaminergic-mediated refinement of spinal locomotor activity remains elusive. This study demonstrates, in larval zebrafish, that increased expression of all D2-like dopamine receptor subtypes, levels of dopamine, and activity of diencephalic dopaminergic neurons correlate with the refinement of locomotor activity.

## Introduction

Throughout life all vertebrates acquire and improve gross motor skills. This is particularly evident in the locomotor system where motor output is initially coarse and becomes progressively more refined during development. For example, a developmental transformation that refines locomotor activity in larval zebrafish occurs between 3 and 4 d postfertilization (dpf; Buss and Drapeau, 2001; Brustein et al., 2003; Lambert et al., 2012). Swimming in larval zebrafish is composed of discrete episodes separated by periods of inactivity (Buss and Drapeau, 2001). At an immature stage of development (3 dpf), larval zebrafish are mostly inactive, and swimming is composed of erratic, long duration (∼1,000 ms) episodes. In contrast, at a more mature stage of development (≥4 dpf), larval zebrafish are more active, and swimming is composed of goal-directed, short duration (∼250 ms) episodes. Our previous work demonstrated that dopaminergic signaling through inhibitory D2-like receptor (D2-likeR) subtypes (D2R, D3R, D4R), specifically D4R, was necessary for the developmental transformation of this behaviorally relevant locomotor activity (Lambert et al., 2012). However, an understanding of the molecular and functional mechanisms underlying the dopaminergic-mediated refinement of spinal locomotor activity remains elusive.

In vertebrates, the dopaminergic system has been associated with movement disorders (Hening et al., 2004; Kordower et al., 2013; Shetty et al., 2019; Pagiazitis et al., 2025), the modulation of spinal locomotor circuits (Svensson et al., 2003; Miles and Sillar, 2011; Clemens et al., 2012; Sharples et al., 2014), Willis–Ekbom disease/restless legs syndrome (Ondo et al., 2000; Clemens et al., 2006), and motor skill learning (Krakauer and Mazzoni, 2011; Wolpert et al., 2011). Most studies on the dopaminergic system demonstrate that dopaminergic neurons located in the midbrain and ventral tegmental area, defined as anatomical groups A8-10 in mammals, are functionally altered in Parkinson's disease and addiction (Björklund and Dunnett, 2007). Interestingly, the diencephalic A11 group of dopaminergic neurons provides the sole source of spinal dopaminergic innervation in mammals (Skagerberg and Lindvall, 1985).

In zebrafish, far-projecting diencephalic dopaminergic neurons (DDNs), homologous to the A11 group in mammals, are located in the ventral diencephalic posterior tubercular (PT) area, which is anatomically delineated into the anterior rostral (PTar) and anterior caudal (PTac) groups (Ryu et al., 2007; Tay et al., 2011). In mature larvae, spontaneous DDN activity, monitored with in vivo calcium imaging, is synchronized both within and between PTar and PTac groups and is correlated with locomotor and mechanosensory activity (Reinig et al., 2017). Further, PTar DDNs in mature larvae are tonically active at rest and have a phasic bursting activity pattern during locomotor activity (Jay et al., 2015). However, it is not known if activity in the DDNs changes during development.

The DDNs project to the spinal cord forming the dopaminergic diencephalospinal tract (DDT), which is considered the most conserved part of the dopaminergic system (Takada et al., 1988; Ryu et al., 2007; Kastenhuber et al., 2010). The DDT is developmentally defined in both zebrafish and mouse by the homeodomain transcription factors, Orthopedia 1a and 1b (Otpa and Otpb; Ryu et al., 2007). The DDT is present in the spinal cord as early as 1 dpf and projects along the rostrocaudal extent of the cord by 3 dpf (Kastenhuber et al., 2010). Interestingly, the presence of dopamine transporter and tyrosine hydroxylase in the DDNs at 3 dpf implies that dopamine is available for release (Xi et al., 2011). Therefore, coarse swimming produced by immature larvae (e.g., long duration episodes) could be due, in part, to a lack of functional D2-likeRs present in locomotor-related spinal neurons, a lack of endogenous dopamine release in the spinal cord, a lack of activity in the DDNs, or a combination of these. The work presented here provides evidence that dopamine receptors are functional in spinal circuits of immature larvae and that increases in D2-likeR transcript levels, dopamine level, and DDN activity correlate with the developmental transformation of locomotor activity.

## Materials and Methods

### Zebrafish lines and care

The University of Minnesota Institutional Animal Care and Use Committee approved all protocols used in this study. Adult zebrafish diet and recirculating system water values were described previously (Wahlstrom-Helgren et al., 2019). Experimental animals were housed in a lighted (14/10 h light/dark) 28.5°C incubator in water containing 60 µg/ml Instant Ocean Sea Salt (Instant Ocean) until 7 dpf. Wild-type (WT, Segrest Farms) larvae (3–7 dpf) were used in all electrophysiology, qRT-PCR, and metabolomic experiments. Double transgenic *Tg(th:Gal4^m1233^;UAS:GCaMP6s^nk13a^)* larvae (3–5 dpf) in the Casper (*roy^−/−^ nacre^−/−^*) background were used in all calcium imaging experiments.

### Spinal transections

Larvae were anesthetized and spinal transections were made with a fine razorblade (FA-10 Feather S, Ted Pella) held with a blade breaker and holder (10053-09, Fine Science Tools). Spinalized preparations were generated in WT at 3 dpf by transecting the nervous system between body segments 3 and 4, just caudal to the hindbrain–spinal cord junction. Spinalization completely separated the brain from the spinal cord, ensuring that all descending inputs to the transected spinal cord were eliminated.

### NMDA-induced fictive swimming—peripheral nerve recording

**Figure 1.**
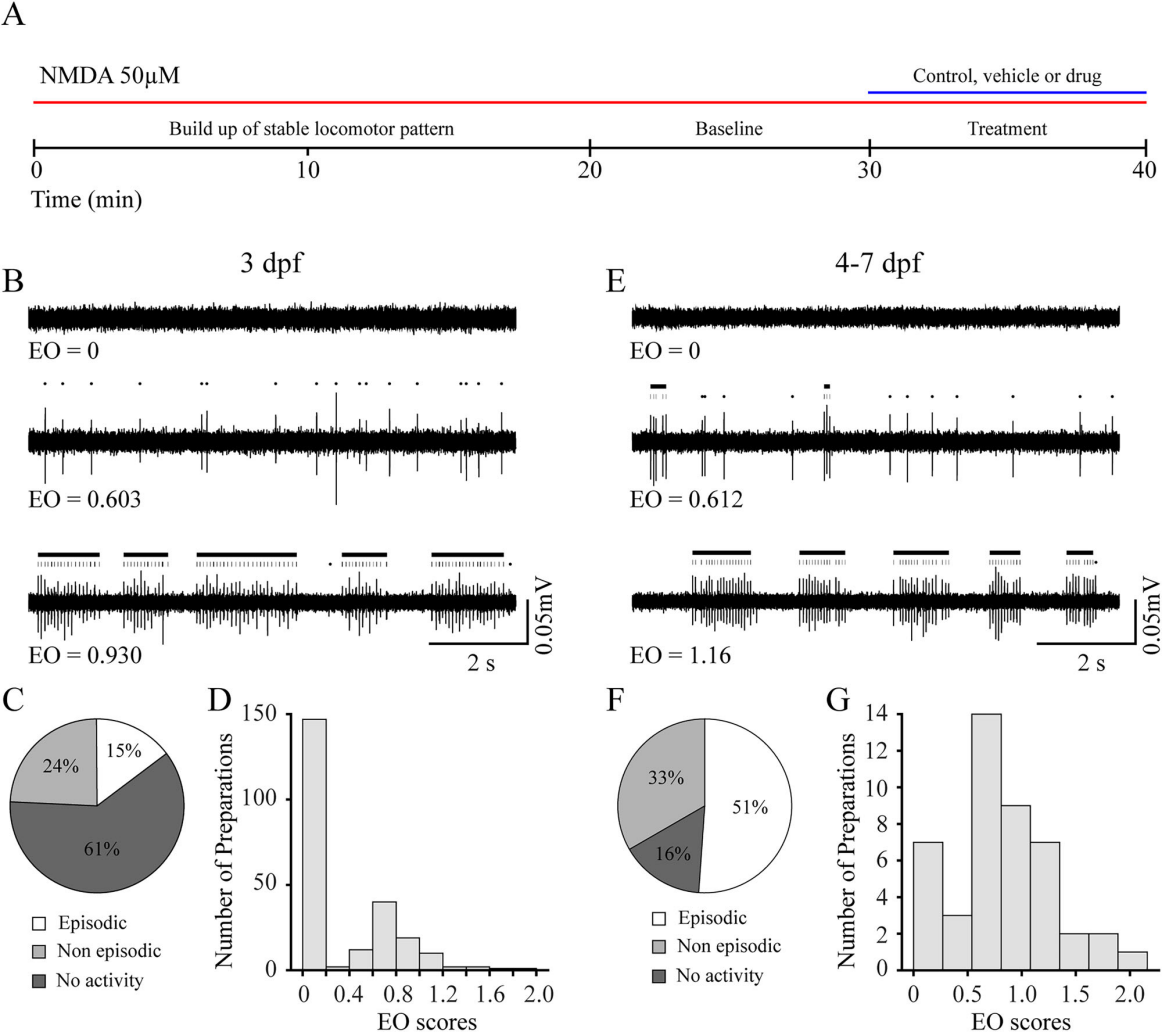
Episodically organized NMDA-induced fictive swimming is less prevalent in immature larvae. ***A***, Experimental paradigm. NMDA was applied to the bath for the duration of the experiment (red line). During the first 20 min, the locomotor pattern built up and became stable. The baseline period started at 20 min and locomotor properties were measured during the last 2 min of the 10 min baseline period. Treatment locomotor properties were measured during the last 2 min of the 10 min treatment period. ***B***, Representative peripheral nerve recordings of fictive locomotor activity from immature (3 dpf) larvae demonstrate three outcomes: no activity (top trace), nonepisodic activity (middle trace), and episodic activity (bottom trace). Black dots above the middle and bottom traces indicate peripheral nerve bursts not organized into episodes, while vertical black lines indicate peripheral nerve bursts organized into episodes. Black horizontal bars indicate episodes. Values for episodic organization (EO) are indicated below the traces. ***C***, Pie chart showing the proportion [15% (35 of 236); white] of immature larvae generates episodically organized (EO ≥ 0.75) locomotor activity. ***D***, Frequency histogram showing the distribution of EO scores for immature larvae. ***E***, Representative peripheral nerve recordings of fictive locomotor activity from mature (4–7 dpf) larvae demonstrate three outcomes: no activity (top trace), nonepisodic activity (middle trace), and episodic activity (bottom trace). Black dots above the middle and bottom traces indicate peripheral nerve bursts not organized into episodes, while vertical black lines indicate peripheral nerve bursts organized into episodes. Black horizontal bars indicate episodes. Values for episodic organization (EO) are indicated below the traces. ***F***, Pie chart showing the proportion [51% (23 of 45); white] of mature larvae generates episodically organized (EO ≥ 0.75) locomotor activity. ***G***, Frequency histogram showing the distribution of EO scores for mature larvae.

Established procedures for peripheral nerve recordings were used (Masino and Fetcho, 2005; Lambert et al., 2012). Briefly, larvae were anesthetized with 0.02% Tricaine-S in extracellular saline composed of the following (in mM): 134 NaCl, 2.9 KCl, 1.2 MgCl_2_, 2.1 CaCl_2_, and 10 HEPES, adjusted to pH 7.8 with NaOH (10 N) and adjusted to 290–300 mOsm/L with sucrose. Larvae were pinned laterally through the notochord to a dissecting dish and subsequently spinalized. Next, the skin was removed with forceps to expose peripheral nerves, and α-bungarotoxin (25 µM in extracellular saline; 2133, Tocris) was applied for 10 min to induce paralysis. Dissecting dishes were placed on a compound microscope (BX51 WI, Olympus) and the preparations were perfused with extracellular saline that contained NMDA (50 µM) at a rate of 0.4 ml/min in a volume of ∼1–2 ml for the duration of the experiment to elicit NMDA-induced fictive swimming (Fig. 1). Glass suction electrodes were positioned on the peripheral nerves at intermyotomal clefts, and recordings were initiated after 10–15 min of perfusion. Voltage signals were amplified with an Axon MultiClamp 700B amplifier connected to a Digidata 1440A digitizer. Signals were sampled at 10 kHz, bandpass filtered to 100–1,000 Hz, and recorded using pClamp 10 software (Molecular Devices).

Peripheral nerve recordings were analyzed by an in-house program written in MATLAB (The MathWorks) to measure properties of fictive swimming described previously (Wiggin et al., 2012). NMDA-induced locomotor properties were analyzed at two timepoints: baseline properties were measured during a 2 min window at the end of the end of the baseline 10 min period of stable locomotor activity, and treatment (control, vehicle, or drug application) properties were measured during a 2 min window at the end of the treatment 10 min period of stable locomotor activity (Fig. 1*A*). Episodic organization (EO) scores were calculated, as described previously (Wiggin et al., 2012; Montgomery et al., 2021). Briefly, EO was measured as the log_10_ ratio of the means of long interburst periods to short interburst periods. Short (interepisode-like) and long (intra-episode-like) interburst periods were determined using a critical value of 2 standard deviations above the mean of the burst periods for each preparation. Higher EO scores represent a large difference between these means, such that bursts are organized into discrete episodes. Lower EO scores represent a small difference between these means, such that bursts are less organized into discrete episodes. An EO score of zero (0) represented experimental preparations that either generated no locomotor activity or that had <50 total bursts within the 2 min analysis window. The analysis of pharmacological perturbation experiments was restricted to preparations that produced an EO score at baseline of ≥0.75 (Fig. 1) and that had >200 bursts within the 2 min analysis window. Episodes were defined as collections of at least three peripheral nerve bursts induced by NMDA separated by <150 ms of quiescence between bursts (Wiggin et al., 2012). Total burst number was defined as the count of peripheral nerve bursts recorded during the 2 min analysis window. Episode duration was defined as the time of onset of the first burst of an episode until the offset of the final burst of the same episode. All the episode durations within the 2 min analysis window were averaged to find the mean episode duration for each timepoint. Mean bursts per episode was defined as the average of the count of peripheral nerve bursts within all episodes during the 2 min analysis window. Mean burst duration was defined as the time of onset of the burst to its offset and averaged for all bursts within episodes during the 2 min analysis window. Mean burst frequency was defined as the average of the burst frequencies, the inverse of the interburst period which is defined for each pair of bursts within episodes as the time from the onset of the first burst to the onset of the second burst, for all bursts within episodes during the 2 min analysis window, excluding the last burst within each episode.

### Pharmacology

Stock solutions of NMDA (10 mM; M3262, Sigma-Aldrich) were dissolved in extracellular saline and diluted in extracellular saline to working concentration (50 µM). Stock solutions of the D2-likeR-specific agonist [quinpirole (3 mM); Q102, Sigma-Aldrich] and D4R-specific agonist (PD168,077 (7.5 mM); P233, Sigma-Aldrich) were dissolved in deionized water or dimethylsulfoxide (DMSO; D5879, Sigma-Aldrich), respectively. Stock solutions were diluted in extracellular saline to the working concentration (quinpirole, 10 µM; PD168,077, 1 µM; DMSO, 0.02%) determined previously (Lambert et al., 2012).

### Reverse transcription quantitative polymerase chain reaction

Larvae were collected at both 3 and 5 dpf from four separate clutches (groups). To separate the heads from the bodies, larvae were anesthetized (0.04% Tricaine, MS-222) and placed on a bed of Sylgard, and transections were performed as described above. Additionally, the yolk sac, yolk finger, and gut were manually removed from the transected bodies. The transected tissue was then placed immediately in saline on ice. The heads and bodies from 200 larvae/group were processed separately for the extraction of total RNA (RNeasy Mini Kit, Qiagen) using routine procedures (Martin et al., 2019; Parvin et al., 2021).

Total RNA (100 ng) was reverse transcribed to produce cDNA using TaqMan EZ RT-PCR Core reagents (Applied Biosystems). The expression levels of D2-like receptor transcripts (*drd2a*, *drd2b*, *drd2l*, *drd3*, *drd4a*, *drd4b*, *drd4rs*; current nomenclature) were measured by TaqMan quantitative real-time (qRT)-PCR using a 7900HT Fast Real-Time PCR System (Applied Biosystems), as replicates (×2). β*-actin* served as a reference gene. Validated qPCR assays (probes, forward and reverse primers) were used for the following genes: *drd2a* (Assay ID Dr03106158_m1), *drd2b* (Assay ID Dr03093765_m1), *drd2l* (Assay ID Dr03119255_m1), *drd3* (Assay ID Dr03131905_m1), *drd4a* (Assay ID Dr03090314_m1), *drd4b* (Assay ID Dr03096340_m1), *drd4-rs* (Assay ID Dr03096342_m1), and *actin* (Assay ID Dr03432610_m1). All assays were obtained from Thermo Fisher Scientific. The targeted gene accession numbers from NCBI are as follows: *drd2a* (NM_183068.1), *drd2b* (NM_197936.1), *drd2l* (NM_197935.1), *drd3* (Dr03131905_m1), *drd4a* (NM_001012616.3), *drd4b* (NM_001012618.1), *drd4-rs* (NM_001012620.3), and *actin* (NM_131031.2). The delta-delta Ct method (2^(Δ-Δ CT)^) was used to measure relative changes in gene expression. The expression for each gene of interest was normalized to β*-actin* expression and presented as a fold change, increase or decrease, in 3 dpf groups, which were normalized to 1 and compared with 5 dpf groups.

### Liquid chromatography-mass spectrometry

Samples of intact zebrafish were collected from three separate clutches on days 3 and 5 postfertilization. Each of the samples was reconstituted in lysis buffer (210 µl; 97.8% water, 2% acetonitrile, and 0.2% formic acid) and ^13^C_6_-dopamine (2 ng; CLM-3369-PK, Cambridge Isotope Laboratories) and transferred to Precellys homogenization tubes (500 µl; 432-0293, Avantor). Next, samples were subjected to three, 30 s pulses of homogenization at 5,800 revolutions per minute (rpm) in a Precellys Evolution homogenizer (P002511-PEVT0-A.0, Betin). Following homogenization, supernatant (10 µl) was removed from each sample and used to quantify protein via Bradford Assay (23200, Thermo Fisher Scientific) to normalize dopamine content to total protein. To the remaining samples, methanol (200 µl) was added and the homogenization repeated in the Precellys homogenizer using the same settings. Following the second homogenization, samples were centrifuged at 13,200 rpm for 15 min at room temperature. The supernatant from each sample was then transferred to a 1.7 ml microcentrifuge tube (022363204, Eppendorf) after which chloroform (800 µl) was added to samples. The samples were vortexed briefly, shaken at 600 rpm, incubated at room temperature for 10 min, and centrifuged again for 15 min at 13,200 rpm at room temperature. The aqueous upper layer of each sample was removed and transferred to a new 1.7 ml microcentrifuge tube (022363204, Eppendorf) and dried overnight in a SpeedVac SPD210 (Thermo Fisher Scientific). Samples were then reconstituted in water (90 µl) with 0.1% formic acid for analysis.

For dopamine (36532, Cayman Chemical) quantitation, a calibration curve was constructed with dopamine concentrations of 0, 0.1, 0.5, 1, 5, 10, 50, and 100 ng/ml in water with 0.1% formic acid and a constant ^13^C_6_-dopamine (Cambridge Isotope Laboratories) concentration of 100 ng/ml. Next, samples were analyzed on a mass spectrometer (Sciex QTrap 6500+, SCIEX) in positive mode interfaced with a Waters Acquity UPLC plumbed with a Waters Acquity UPLC BEH C18 Column (130 Å, 1.7 µm, 2.1 mm × 50 mm; Waters). Mass spectrometer method settings included a curtain gas flow of 25 pounds per square inch (psi), a collision gas value of medium, an ion spray voltage of 5,500 V, a temperature of 350°C, an ion source gas 1 value of 20 psi, and an ion source gas 2 value of 30 psi. Mobile phase A consisted of water with 0.1% formic acid, while mobile phase B consisted of acetonitrile with 0.1% formic acid. Samples were run on a 15 min method at 0.2 ml/min with a solvent gradient consisting of 98% A from 0 to 0.1 min, 98–65% A from 0.1 to 5 min, 65–2% A from 5 to 11 min, 2% A from 11 to 12 min, 2–98% A from 12 to 13.5 min, and 98% A from 13.5 to 15 min. Each sample was run four times with fresh aliquots (20 µl) injected for each run. Raw data was analyzed using the Skyline suite (https://skyline.ms/project/home/begin.view).

### In vivo calcium imaging—longitudinal assay

Transgenic zebrafish larvae (*th:Gal4^m1233^;UAS:GCaMP6s^nk13a^*) were anesthetized with 0.02% Tricaine-S (Western Chemical) in extracellular saline and embedded (positioned dorsal ventrally) in 0.2% low melting point agarose. Dissecting dishes were placed on a (BX51WI, Olympus) compound microscope and perfused with extracellular saline at a rate of 0.4 ml/min in a volume of ∼1–2 ml with recordings initiated after 20–30 min of perfusion. GCaMP signal was visualized using epifluorescent blue light (1.9 mW/cm^2^; X-Cite 12-LED Boost lamp, Excelitas Technologies) and 20×/NA 0.5 water immersion objective (UMPlanFL N, Olympus) and a fluorescence imaging camera (Retiga EXi, QImaging). Calcium signals were collected at 5 Hz for 30 min. Subsequently, each larva was unembedded and returned to embryo water and in vivo calcium imaging was repeated at 3 and 5 dpf.

Anatomical atlases were used as guides to identify bilateral DDN/PTac neurons (Reinig et al., 2017; Haehnel-Taguchi et al., 2018). Bilateral ROIs were placed over regions of identified DDN/PTac neurons (Fig. 8*A–D*). To control for background signal, an equal sized ROI was placed over a region devoid of indicator and neurons. Calcium signals were bleach corrected using Fiji (Schindelin et al., 2012) and changes in fluorescence (Δ*F*/*F*) were measured using Microsoft Excel (Microsoft Corporation). The “findpeaks” function in MATLAB was used to find the number of peaks and the magnitude of each peak. The minimum interval between peaks was set to 5 frames (1 s) and the minimum Δ*F*/*F* prominence was set to 0.35. To determine the average power of the calcium signals for each recording, the root mean square of the signals (Δ*F*/*F*) was determined and squared.

### Statistical analysis

All statistical analyses were performed with SigmaPlot 16.0 (Graffiti). Episodic properties of locomotor activity, measured with peripheral nerve recordings, were analyzed using *z*-tests, Student's *t* tests, or paired *t* tests for parametric data and Mann–Whitney rank sum test or Wilcoxon signed rank test for nonparametric data. Calcium transients recorded from bilateral DDN/PTac neuron groups were analyzed using Pearson's Product Moment Correlation and Student's *t* test for parametric data and Mann–Whitney rank sum test for nonparametric data. qRT-PCR of D2-likeR mRNA expression between 3 and 5 dpf larvae were analyzed using Student's *t* test with Prism v9 (GraphPad Software). Metabolomic analysis of DA levels were analyzed using Student’s *t* test (Origin 2025 Software). All experimenters were blinded to age for qRT-PCR and metabolomic analysis. Significance was established using an α criterion of *p* = 0.05. In the figures, *p < 0.05, **p < 0.01, and ***p < 0.001. Data are expressed as means and standard deviation [mean (SD)]. All statistical measures were compiled into tables except for metabolomic analysis of DA levels which was reported in the text.

## Results

### Spinal locomotor output is less functionally organized in immature larvae

Spontaneous swimming episodes produced by immature larvae are less frequent and of longer duration than those produced by mature larvae, indicating that locomotor activity transforms during larval development (Lambert et al., 2012). To assess potential differences in organization of the spinal locomotor output before and after the transformation of locomotor activity, we compared, in spinalized preparations, NMDA-induced locomotor properties between immature and mature larvae (Fig. 1). The proportion of immature larvae that did not generate NMDA-induced activity (Fig. 1*B*, top trace, *C*,*D*) was significantly greater compared with the proportion of mature larvae (Fig. 1*E*, top trace, *F*,*G*; Table 1). In addition, the proportion of immature larvae that produced episodically organized locomotor activity (Fig. 1*B*, bottom trace, *C*,*D*) was significantly lower compared with mature larvae (Fig. 1*E*, top trace, *F*,*G*; Table 1). There was no difference in the proportion of immature larvae that produced nonepisodically organized locomotor activity (Fig. 1*B*, middle trace, *C*,*D*) compared with mature larvae (Fig. 1*E*, middle trace, *F*,*G*; Table 1).

**Table 1.** Proportions of immature and mature larvae that produce NMDA-induced locomotor responses

	Developmental stage	
	3 dpf	4–7 dpf
No activity (%)	61.0 (144 of 236)	15.6 (7 of 45)
	*z* = 5.61	
	*p* < 0.001	
Nonepisodic (%)	24.2 (57 of 236)	33.3 (15 of 45)
	*z* = 1.29	
	*p* = 0.196	
Episodic (%)	14.8 (35 of 236)	51.1 (23 of 45)
	*z* = 5.51	
	*p* < 0.001	

Next, we performed an initial analysis which included all preparations that generated NMDA-induced locomotor activity with an EO score greater than zero (0; Fig. 1*B*,*E*, middle and bottom traces). The EO scores produced by immature larvae were significantly lower compared with mature larvae (Table 2, episodic organization, EO > 0). No other measures of episodic or bursting activity in immature larvae were significantly different compared with mature larvae (Table 2).

**Table 2.** Comparison of NMDA-induced locomotor activity measures between immature and mature larvae

			Developmental stage	
			3 dpf	4–7 dpf
Episodic organization (EO score)	EO > 0	Mean (SD), *n*	0.806 (0.278), 89	0.968 (0.401), 38
		Mann-Whitney *U*	1,289	
		*p*	0.034	
	EO ≥ 0.75	Mean (SD), *n*	0.960 (0.192), 35	1.10 (0.267), 22
		Mann-Whitney *U*	263	
		*p*	0.046	
Mean episode duration (s)	EO > 0	Mean (SD), *n*	1.08 (0.879), 58	0.944 (0.840), 33
		Mann-Whitney *U*	802	
		*p*	0.201	
	EO ≥ 0.75	Mean (SD), *n*	1.13 (0.818), 35	0.870 (0.556), 22
		Mann-Whitney *U*	316	
		*p*	0.258	
Total bursts (n)	EO > 0	Mean (SD), *n*	757 (436), 89	730 (398), 38
		Mann Whitney *U*	1,657	
		*p*	0.858	
	EO ≥ 0.75	Mean (SD), *n*	950 (250), 35	800 (301), 22
		*t*	2.05	
		df	55	
		*p*	0.0457	
Mean bursts per episode (n)	EO > 0	Mean (SD), *n*	15.1 (11.8), 58	13.8 (10.7), 33
		Mann-Whitney *U*	840	
		*p*	0.334	
	EO ≥ 0.75	Mean (SD), *n*	16.5 (12.1), 35	13.9 (9.05), 22
		Mann-Whitney *U*	344	
		*p*	0.502	
Mean burst duration (ms)	EO > 0	Mean (SD), *n*	7.14 (2.02), 58	6.69 (2.40), 33
		Mann-Whitney *U*	799	
		*p*	0.192	
	EO ≥ 0.75	Mean (SD), *n*	6.79 (1.54), 35	6.64 (2.23), 22
		Mann-Whitney *U*	322	
		*p*	0.302	
Mean burst frequency (Hz)	EO > 0	Mean (SD), *n*	14.0 (2.69), 58	14.4 (2.73), 33
		Mann-Whitney *U*	872	
		*p*	0.483	
	EO ≥ 0.75	Mean (SD), *n*	14.2 (2.44), 35	15.0 (2.78), 22
		Mann-Whitney *U*	318	
		*p*	0.272	

Values reported as mean (SD).

Finally, we restricted our analysis to only preparations that produced NMDA-induced locomotor activity with an EO score >0.75 (Fig. 1*B*,*E*, bottom traces). Although episodes generated by immature larvae were well organized, the EO scores were significantly lower (Table 2, episodic organization, EO > 0.75), and the total number of bursts were significantly higher compared with mature larvae (Table 2, total bursts, EO > 0.75). All other measures of episodic and bursting activity were not significantly different in immature compared with mature larvae (Table 2). While the spinal locomotor output in immature larvae was less functionally organized compared with mature larvae, our results demonstrated that it was sufficiently functional in, at least, a proportion of immature larvae to produce episodically organized locomotor activity.

### D4Rs are functional in immature larvae

Our previous work demonstrated that dopaminergic signaling through inhibitory D2-likeRs was necessary to maintain the mature locomotor phenotype (short duration episodes) following the developmental transformation of locomotor activity (Lambert et al., 2012). Thus, we reasoned that the coarse locomotor pattern produced by immature larvae could be due to a lack of functional D2-likeRs in locomotor-related spinal neurons.

**Figure 2.**
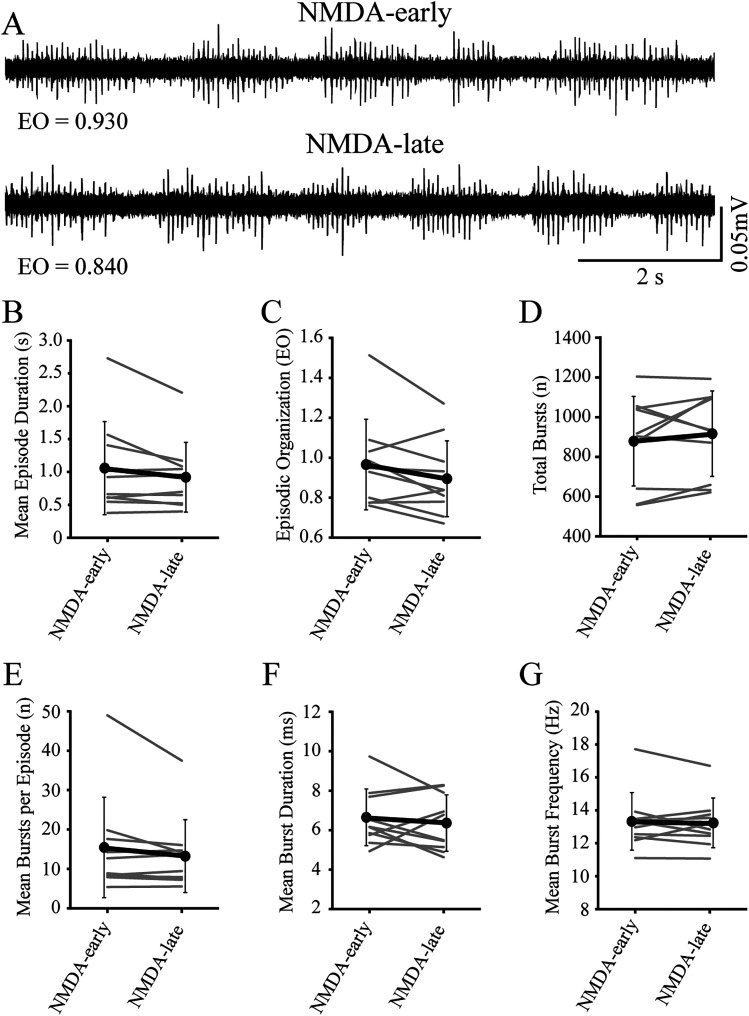
NMDA-induced spinal locomotor activity remains episodically organized and stable in immature larvae. ***A***, Representative peripheral nerve recordings from an individual preparation acquired at early (NMDA-early) and late (NMDA-late) timepoints during NMDA-induced locomotor activity. Values for episodic organization (EO) are indicated below the traces. ***B***, Episode duration is not different between NMDA-early and NMDA-late timepoints. Gray lines indicate within-subjects repeated measures, while black circles represent the mean (SD) (*n* = 10 larvae). ***C***, Episodic organization is not different between NMDA-early and NMDA-late timepoints. Gray lines indicate within-subjects repeated measures, while black circles represent the mean (SD) (*n* = 10 larvae). ***D***, The total number of bursts are not different between NMDA-early and NMDA-late timepoints. Gray lines indicate within-subjects repeated measures, while black circles represent the mean (SD) (*n* = 10 larvae). ***E***, The mean number of bursts per episode is not different between NMDA-early and NMDA-late timepoints. Gray lines indicate within-subjects repeated measures, while black circles represent the mean (SD) (*n* = 10 larvae). ***F***, The mean duration of bursts is not different between NMDA-early and NMDA-late timepoints. Gray lines indicate within-subjects repeated measures, while black circles represent the mean (SD) (*n* = 10 larvae). ***G***, The mean burst frequency is not different between NMDA-early and NMDA-late timepoints. Gray lines indicate within-subjects repeated measure, while black circles represent the mean (SD) (*n* = 10 larvae).

First, we assessed potential time-dependent (early vs late) effects on the episodic properties of NMDA-induced locomotor activity in immature larvae (Fig. 2*A*). No significant differences were found for episodic or bursting measures (Fig. 2*B–G*; Tables 3, 4, respectively) in control preparations (NMDA-only). These results demonstrated that NMDA-induced activity remained episodically organized and stable for the duration of the experiment.

**Table 3.** Comparison of episodic locomotor properties in immature larvae under different experimental conditions

	Mean episode duration (s)		Episodic organization (EO score)		Total bursts (*n*)	
	Baseline	Treatment	Baseline	Treatment	Baseline	Treatment
	Mean (SD)	Mean (SD)	Mean (SD)	Mean (SD)	Mean (SD)	Mean (SD)
Control	1.04 (0.705)	0.924 (0.526)	0.961 (0.227)	0.898 (0.190)	880 (224)	913 (214)
	*Z* = −1.07		*t* = 1.90		*t* = −0.951	
	*p* = 0.322		df = 9		df = 9	
	*n* = 10		*p* = 0.0900		*p* = 0.367	
			*n* = 10		*n* = 10	
D2-likeR agonist	0.936 (1.14)	0.523 (0.582)	0.841 (0.0722)	0.645 (0.110)	800 (282)	695 (372)
	*Z* = −2.02		*t* = 3.98		*t* = 1.16	
	*p* = 0.063		df = 4		df = 4	
	*n* = 5		*p* = 0.0164		*p* = 0.312	
			*n* = 5		*n* = 5	
D4R agonist	2.03 (0.651)	1.46 (0.604)	1.11 (0.134)	0.894 (0.123)	1,234 (129)	1,219 (165)
	*t* = 3.14		*t* = 5.27		*t* = 0.448	
	df = 6		df = 6		df = 6	
	*p* = 0.0202		*p* = 0.00188		*p* = 0.670	
	*n* = 7		*n* = 7		*n* = 7	
DMSO	0.668 (0.540)	0.479 (0.356)	0.937 (0.105)	0.813 (0.0683)	817 (263)	840 (284)
	*Z* = −2.02		*t* = 2.07		*t* = −0.506	
	*p* = 0.063		df = 4		df = 4	
	*n* = 5		*p* = 0.107		*p* = 0.639	
			*n* = 5		*n* = 5	

Values reported as mean (SD).

**Table 4.** Comparison of bursting locomotor properties in immature larvae under different experimental conditions

	Mean bursts per episode (*n*)		Mean burst duration (ms)		Mean burst frequency (within episodes) (Hz)	
	Baseline	Treatment	Baseline	Treatment	Baseline	Treatment
	Mean (SD)	Mean (SD)	Mean (SD)	Mean (SD)	Mean (SD)	Mean (SD)
Control	15.2 (12.7)	13.3 (9.24)	6.61 (1.44)	6.38 (1.43)	13.3 (1.75)	13.2 (1.51)
	*Z* = −1.27		*t* = 0.598		*t* = 0.394	
	*p* = 0.232		df = 9		df = 9	
	*n* = 10		*p* = 0.564		*p* = 0.703	
			*n* = 10		*n* = 10	
D2-likeR agonist	12.1 (12.6)	6.97 (6.25)	7.34 (1.29)	7.12 (1.83)	13.14 (1.69)	13.23 (2.89)
	*Z* = −2.02		*t* = 0.580		*Z* = −0.674	
	*p* = 0.063		df = 4		*p* = 0.625	
	*n* = 5		*p* = 0.593		*n* = 5	
			*n* = 5			
D4R agonist	30.7 (9.80)	20.9 (8.72)	6.89 (1.58)	6.88 (1.72)	14.9 (0.580)	14.1 (0.766)
	*t* = 3.50		*t* = 0.0213		*t* = 3.25	
	df = 6		df = 6		df = 6	
	*p* = 0.0128		*p* = 0.984		*p* = 0.0175	
	*n* = 7		*n* = 7		*n* = 7	
DMSO	10.4 (7.30)	7.74 (4.67)	6.17 (0.933)	6.16 (1.16)	15.3 (1.98)	15.5 (2.27)
	*t* = 2.13		*t* = 0.0206		*Z* = −0.405	
	df = 4		df = 4		df = 4	
	*p* = 0.100		*p* = 0.985		*p* = 0.813	
	*n* = 5		*n* = 5		*n* = 5	

Values reported as mean (SD).

**Figure 3.**
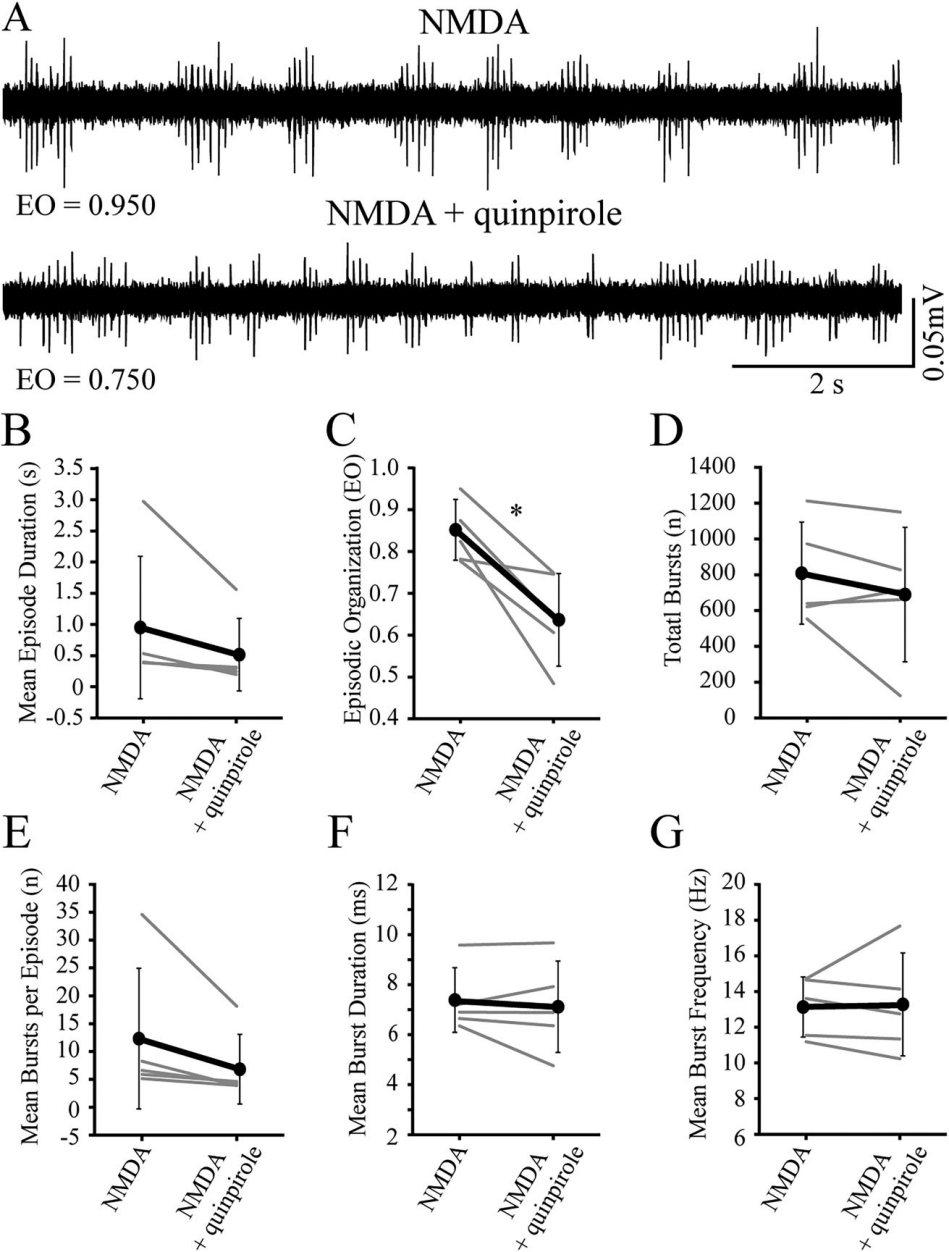
Application of exogenous D2-like receptor subtype agonist during NMDA-induced fictive swimming in immature larvae decreases episodic organization. ***A***, Representative peripheral nerve recordings from an individual preparation acquired at baseline (NMDA) and during agonist treatment (NMDA + quinpirole). Values for episodic organization (EO) are indicated below the traces. ***B***, Episode duration decreases, but not significantly, during treatment (NMDA + quinpirole) compared with baseline (NMDA). Gray lines indicate within-subjects repeated measures, while black circles represent the mean (SD) (*n* = 5 larvae). ***C***, Episodic organization decreases during treatment (NMDA + quinpirole) compared with baseline (NMDA). Gray lines indicate within-subjects repeated measures, while black circles represent the mean (SD) (*n* = 5 larvae). ***D***, The total number of bursts are not affected during treatment (NMDA + quinpirole) compared with baseline (NMDA). Gray lines indicate within-subjects repeated measures, while black circles represent the mean (SD) (*n* = 5 larvae). ***E***, The mean number of bursts per episode is not different between baseline (NMDA) and treatment (NMDA + quinpirole). Gray lines indicate within-subjects repeated measures, while black circles represent the mean (SD) (*n* = 5 larvae). ***F***, The mean duration of bursts is not different between baseline (NMDA) and treatment (NMDA + quinpirole). Gray lines indicate within-subjects repeated measures, while black circles represent the mean (SD) (*n* = 5 larvae). ***G***, The mean burst frequency is not different between baseline (NMDA) and treatment (NMDA + quinpirole). Gray lines indicate within-subjects repeated measures, while black circles represent the mean (SD) (*n* = 5 larvae). Asterisk indicates significant differences at *p* < 0.05.

**Figure 4.**
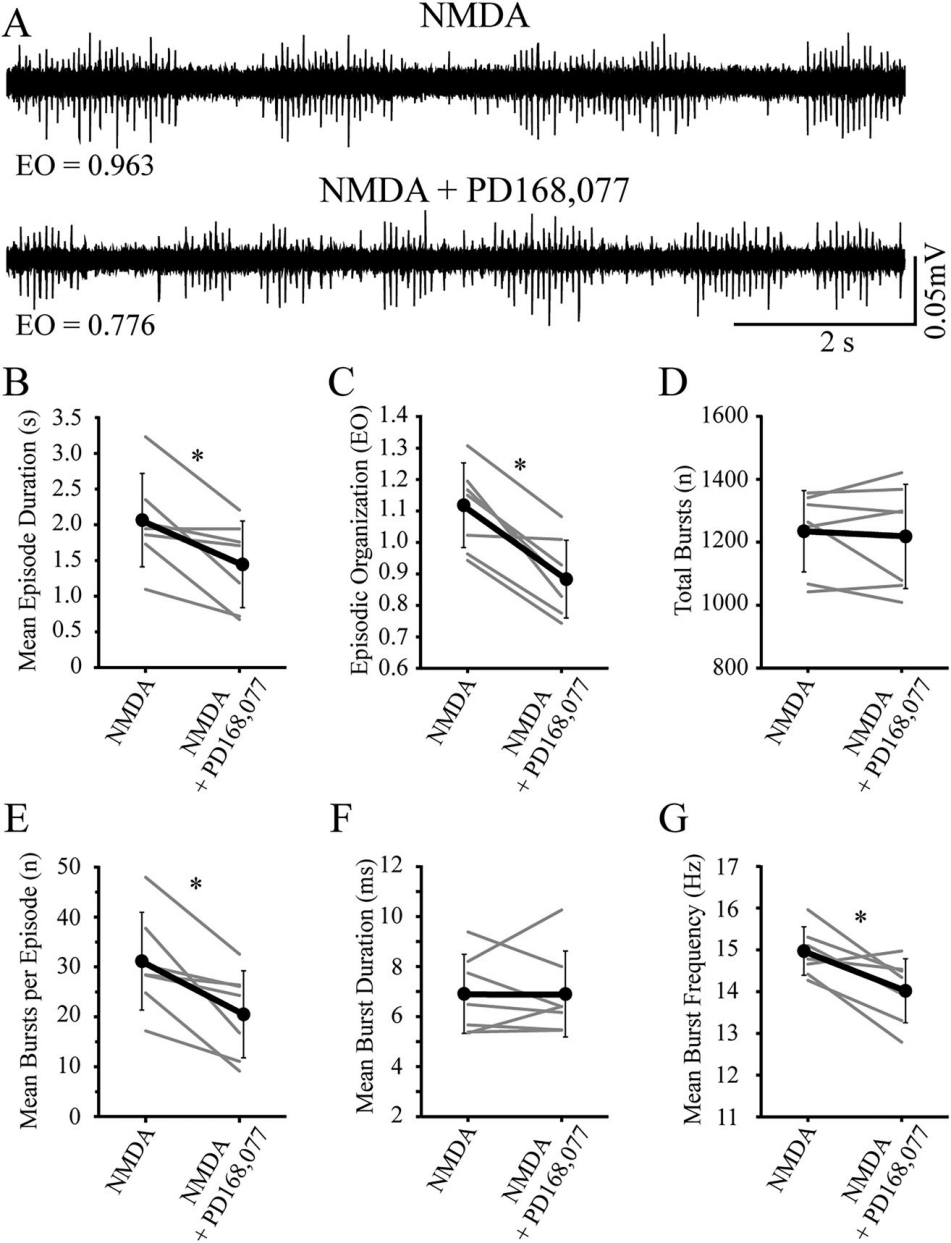
Application of exogenous D4-specific receptor subtype agonist during NMDA-induced fictive swimming in immature larvae decreases episode duration and episodic organization. ***A***, Representative peripheral nerve recordings from an individual preparation acquired at baseline (NMDA) and during agonist treatment (NMDA + PD 168,077). Values for episodic organization (EO) are indicated below the traces. ***B***, Episode duration decreases during treatment (NMDA + PD 168,077) compared with baseline (NMDA). Gray lines indicate within-subjects repeated measures, while black circles represent the mean (SD) (*n* = 7 larvae). ***C***, Episodic organization decreases during treatment (NMDA + PD 168,077) compared with baseline (NMDA). Gray lines indicate within-subjects repeated measures, while black circles represent the mean (SD) (*n* = 7 larvae). ***D***, The total number of bursts are not affected during treatment (NMDA + PD 168,077) compared with baseline (NMDA). Gray lines indicate within-subjects repeated measures, while black circles represent the mean (SD) (*n* = 7 larvae). ***E***, The mean number of bursts per episode decreases during treatment (NMDA + PD 168,077) compared with baseline (NMDA). Gray lines indicate within-subjects repeated measures, while black circles represent the mean (SD) (*n* = 7 larvae). ***F***, The mean duration of bursts is not affected during treatment (NMDA + PD 168,077) compared with baseline (NMDA). Gray lines indicate within-subjects repeated measures, while black circles represent the mean (SD) (*n* = 7 larvae). ***G***, The mean burst frequency decreases during treatment (NMDA + PD 168,077) compared with baseline (NMDA). Gray lines indicate within-subjects repeated measures, while black circles represent the mean (SD) (*n* = 7 larvae). Asterisks indicate significant differences at *p* < 0.05.

Next, to establish that inhibitory dopamine receptors were functional in immature larvae, we compared episodic properties of NMDA-induced locomotor activity before (baseline) and during application (treatment) of D2-likeR (quinpirole) or D4R-specific (PD168,077) agonists (Figs. 3*A*, 4*A*). Application of the D2-likeR agonist produced a significant decrease in EO score (Fig. 3*C*, Table 3) but no significant differences in the other measures of episodic or bursting activity (Fig. 3*B–G*; Tables 3, 4, respectively). Application of the D4R agonist produced significant decreases for mean episode durations (Fig. 4*B*, Table 3), EO score (Fig. 4*C*, Table 3), mean bursts per episode (Fig. 4*E*, Table 4), and mean burst frequency (Fig. 4*G*, Table 4), with no significant differences for other measures (Fig. 4*B–G*; Tables 3, 4).

**Figure 5.**
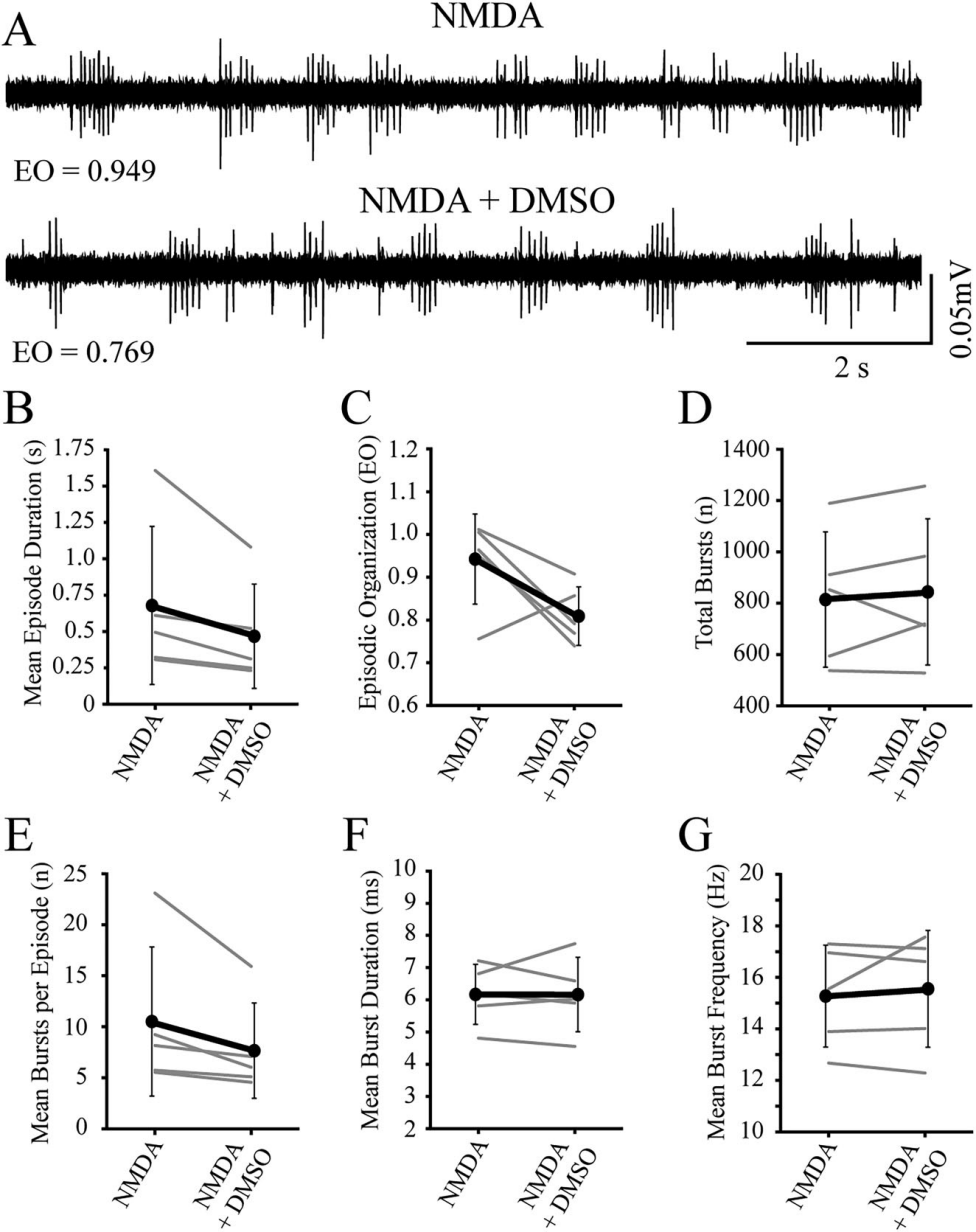
Application of DMSO during NMDA-induced fictive swimming in immature larvae does not affect episode duration, episodic organization, or total number of bursts. ***A***, Representative peripheral nerve recordings from an individual preparation acquired at baseline (NMDA) and during treatment (NMDA + DMSO). Values for episodic organization (EO) are indicated below the traces. ***B***, Episode duration is not affected during treatment (NMDA + DMSO) compared with baseline (NMDA). Gray lines indicate within-subjects repeated measures, while black circles represent the mean (SD) (*n* = 5 larvae). ***C***, Episodic organization is not affected during treatment (NMDA + DMSO) compared with baseline (NMDA). Gray lines indicate within-subjects repeated measures, while black circles represent the mean (SD) (*n* = 5 larvae). ***D***, The total number of bursts are not affected during treatment (NMDA + DMSO) compared with baseline (NMDA). Gray lines indicate within-subjects repeated measures, while black circles represent the mean (SD) (*n* = 5 larvae). ***E***, The mean number of bursts per episode is not different between baseline (NMDA) and treatment (NMDA + DMSO). Gray lines indicate within-subjects repeated measures, while black circles represent the mean (SD) (*n* = 5 larvae). ***F***, The mean duration of bursts burst duration is not different between baseline (NMDA) and treatment (NMDA + DMSO). Gray lines indicate within-subjects repeated measures, while black circles represent the mean (SD) (*n* = 5 larvae). ***G***, The mean burst frequency is not different between baseline (NMDA) and treatment (NMDA + DMSO). Gray lines indicate within-subjects repeated measures, while black circles represent the mean (SD) (*n* = 5 larvae).

Finally, since DMSO was used as the vehicle for the D4R-specific agonist, we controlled for potential effects of vehicle administration on the episodic properties of NMDA-induced locomotor activity (Fig. 5*A*). There were no significant differences in the measures of episodic or bursting activity (Fig. 5*B–G*; Tables 3, 4, respectively) during application of DMSO. These results demonstrated that D4Rs were functional in the spinal locomotor circuits of immature larvae.

### Gene expression of all D2-likeRs is greater in mature larvae

Several lines of evidence suggest that dopamine receptor genes are differentially expressed during development. First, the expression of dopamine receptor genes (*drd1*, *drd2a*, *drd2b*, *drd2l*, *drd3*, and *drd4*) are detected in the brain and/or spinal cord by 2 dpf, and an increase in expression during development (from 2 to 5 dpf) has been described for at least some genes (*drd1* and *drd2b*; Boehmler et al., 2004; Li et al., 2007). Second, we previously reported that the transformation of locomotor activity during development, from long duration swim episodes produced by immature larvae to short duration swim episodes produced by mature larvae, was dependent on D4R signaling (Lambert et al., 2012). Finally, we demonstrated in this study that D4Rs were functional in immature larvae (Figs. 3, 4). However, potential developmental changes in gene expression levels of all D2-likeR subtypes in the spinal cord that function to support this locomotor transformation have not been quantified.

**Figure 6.**
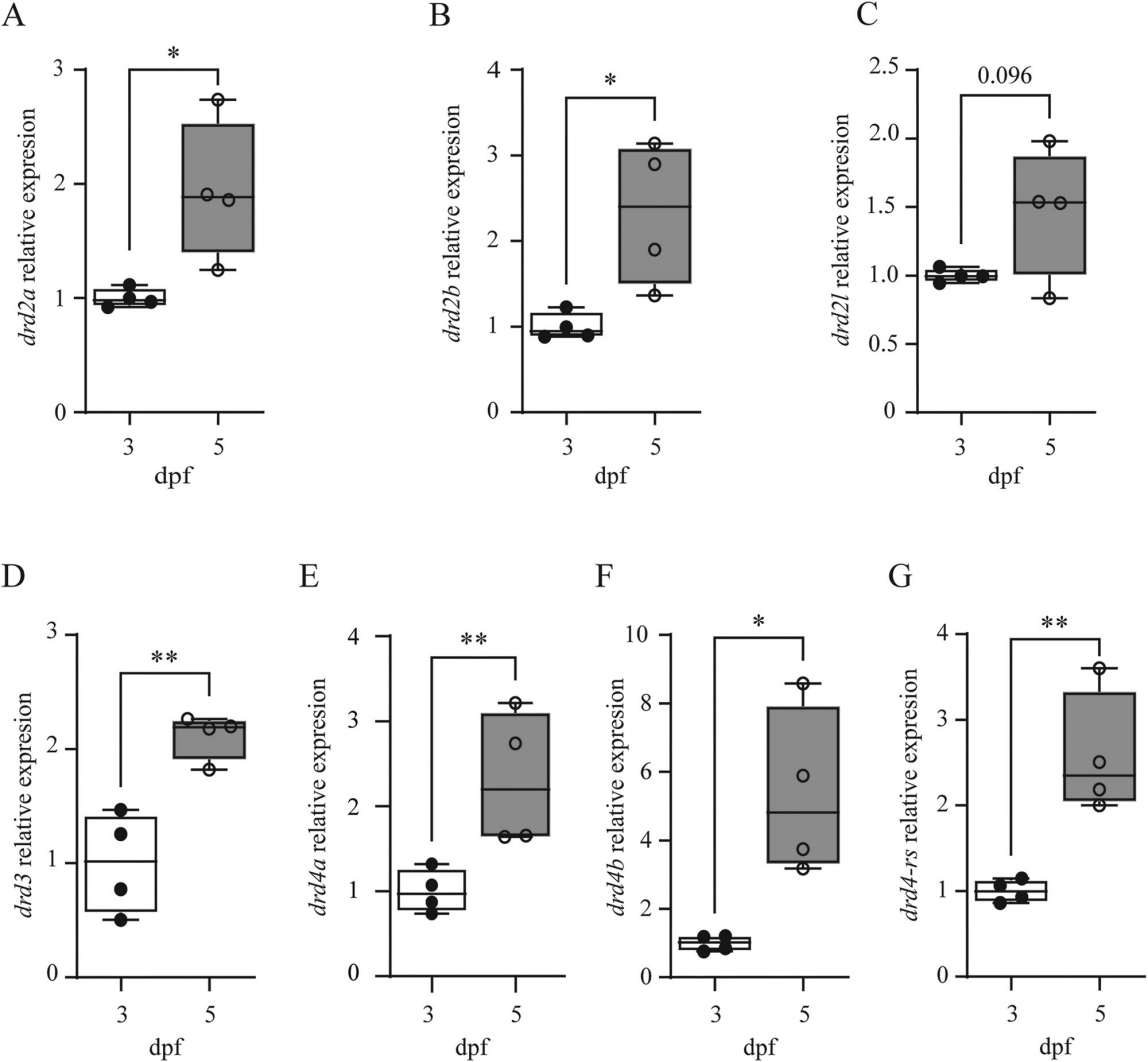
Gene expression of most D2-like receptor subtypes is greater in the transected bodies of mature larvae. ***A–G***, Plots show that expression of all D2-like receptor subtypes, except for *drd2l*, are significantly increased at 5 dpf (open circles) compared with 3 dpf (black-filled circles). Asterisk indicates significant difference at *p < 0.05, **p < 0.01.

Therefore, to compare the levels of gene expression of the D2-likeR subtypes between immature and mature larvae, qRT-PCR was performed on transected bodies as a proxy to analyze mRNA expression in spinal cord. Specifically, we assessed the expression levels of *drd2a*, *drd2b*, *drd2l*, *drd3*, *drd4a*, *drd4b*, and *drd4-rs* mRNA. All mRNA targets, except for *drd2l*, were significantly increased in mature larvae compared with immature larvae (Fig. 6, Table 5). We also assessed mRNA expression of the D2-likeR subtypes in the heads of mature and immature larvae. Consistent with the changes observed in the body, the mRNA levels of all seven D2R targets were significantly increased in mature larvae (data not shown).

**Table 5.** Comparison of mRNA relative expression for all D2-like receptor genes between immature and mature larvae

	Developmental stage	
	3 dpf	5 dpf
Receptor transcript	Mean (SD), *N*	Mean (SD), *N*
*drd2a*	1.00 (0.0825), 4	1.938 (0.612), 4
	*t* = 3.04 df = 6 *p* = 0.0228	
*drd2b*	1.00 (0.158), 4	2.33 (0.835), 4
	*t* = 3.12 df = 6 *p* = 0.0205	
*drd2l*	1.00 (0.0488), 4	1.47 (0.474), 4
	*t* = 1.97 df = 6 *p* = 0.096	
*drd3*	1.00 (0.441), 4	2.12 (0.200), 4
	*t* = 4.61 df = 6 *p* = 0.0037	
*drd4a*	1.00 (0.255), 4	2.31 (0.791), 4
	*t* = 3.16 df = 6 *p* = 0.0196	
*drd4b*	1.00 (0.228), 4	5.35 (2.45), 4
	*t* = 3.53 df = 6 *p* = 0.0124	
*drd4-rs*	1.00 (0.130), 4	2.57 (0.716), 4
	*t* = 4.33 df = 6 *p* = 0.005	

Values are reported as mean (SD).

These results demonstrated that gene expression of all D2-likeR subtypes increased in the bodies of larval zebrafish across the developmental transformation of locomotor activity (Fig. 6). This increase in gene expression in mature larvae is consistent with the hypothesis that locomotor activity is refined by an increase in dopaminergic signaling during development.

### Dopamine levels are greater in mature larvae

Our previous work demonstrated that dopaminergic signaling was required for the developmental transformation of locomotor activity (Lambert et al., 2012), that D4Rs were functional in the spinal locomotor circuits of immature larvae (Figs. 3, 4), and that gene expression of all D2-likeR subtypes increased in the bodies of larval zebrafish across the developmental transformation of locomotor activity (Fig. 6). Thus, we next tested the hypothesis that an increase in DA levels correlated with the transformation of locomotor activity.

**Figure 7.**
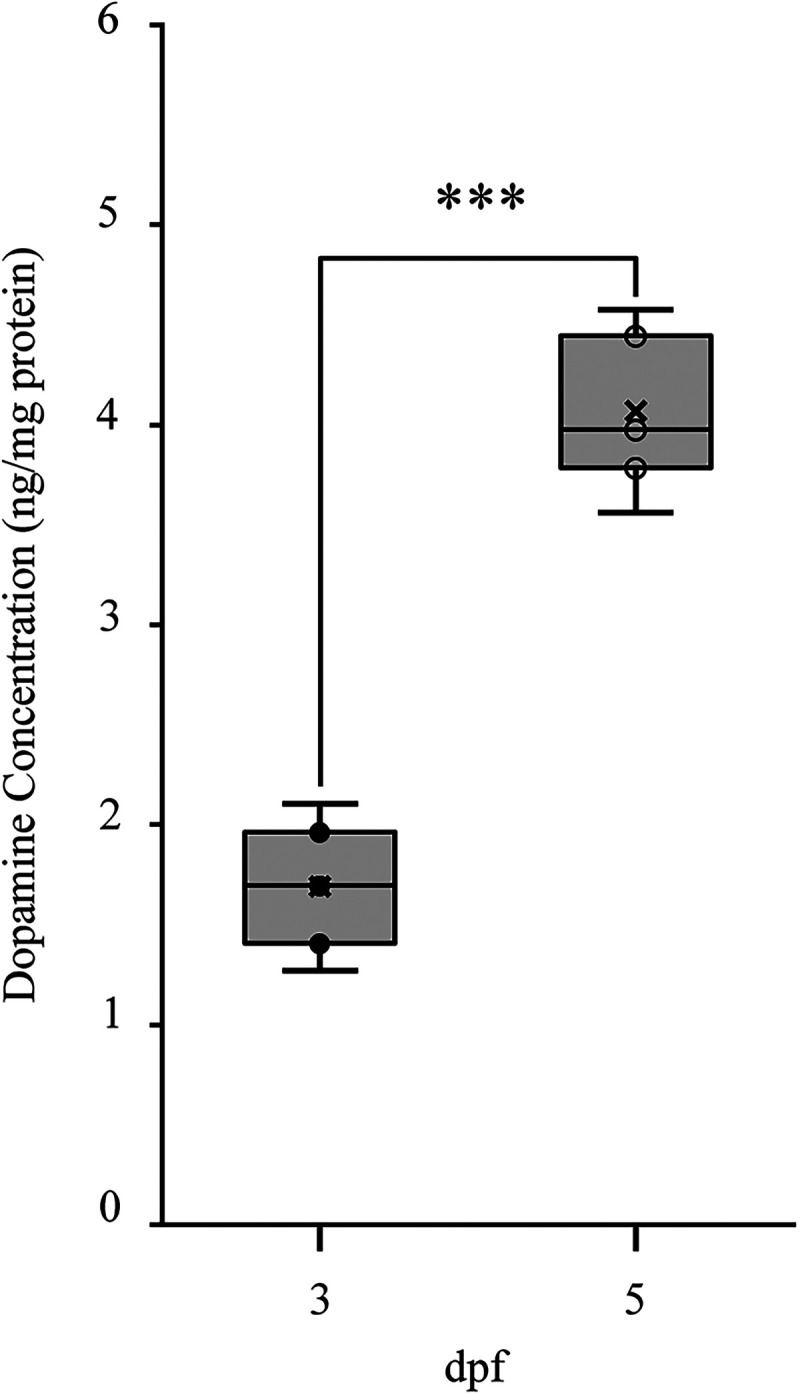
Dopamine levels are greater in mature larvae. Plot shows that the amount of global dopamine from intact larvae are significantly increased at 5 dpf (open circles) compared with 3 dpf (black-filled circles). Asterisk indicates significant difference at ***p < 0.001.

The DDNs in zebrafish, which are located in the ventral diencephalon and homologous to the A11 group in mammals, provide the sole source of dopamine to the spinal cord (Ryu et al., 2007; Tay et al., 2011). Therefore, liquid chromatography-mass spectrometry was performed on intact larvae at 3 and 5 dpf to compare absolute levels of dopamine between immature and mature larvae. Dopamine levels were significantly greater in mature larvae compared with immature larvae [mean (SD): 3 dpf: 1.69 (0.278); 5 dpf 4.07 (0.339); *t* = −9.41, df = 4, *p* = 7.10 × 10^−4^, *N* = 3; Fig. 7]. These results are consistent with the hypothesis that locomotor activity is transformed through an increase in dopaminergic signaling during development.

### DDN activity is greater in mature larvae

Although the level of dopamine in mature larvae is greater than in immature larvae (Fig. 7), direct evidence demonstrating that dopamine is more readily released in the spinal cord of mature larvae is lacking. We hypothesized that increased DDN/PTac activity from the immature to the mature stage of larval development was responsible for locomotor transformation. Thus, we used DDN/PTac activity as a proxy for dopamine release. Since a larger amount of dopamine was present in mature larvae (Fig. 7), it is possible that an increase in activity in DDN/PTac neurons in mature larvae would increase the yield of dopamine release on spinal locomotor circuits.

**Figure 8.**
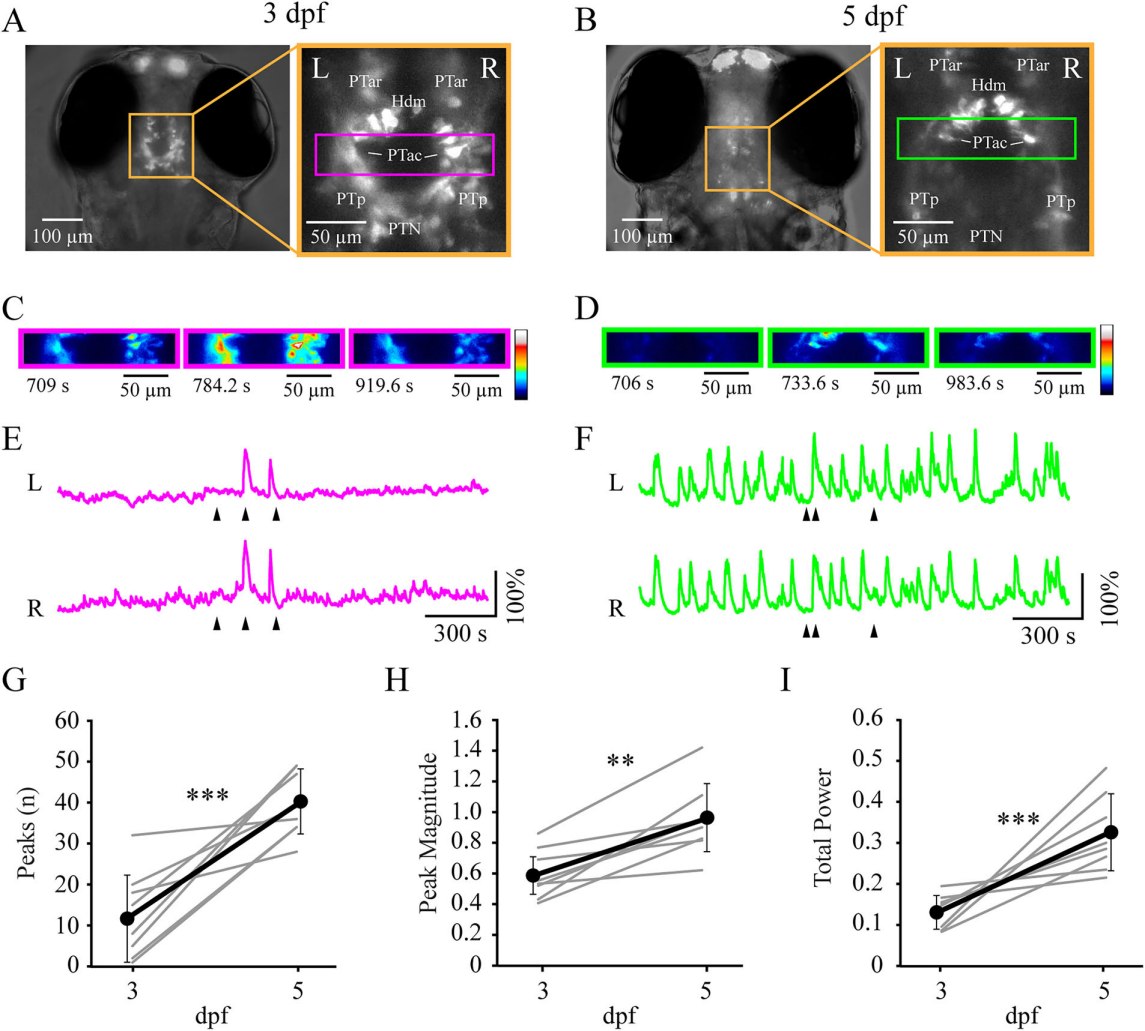
DDN/PTac neuronal activity is greater in mature larvae. ***A***, ***B***, Representative example of an individual transgenic larva (*th:Gal4^m1233^;UAS:GCaMP6s^nk13a^*) expressing GCaMP6s in dopaminergic neurons (orange boxes) imaged at both 3 dpf (***A***) and 5 dpf (***B***). Insets, Orange boxes indicate magnified view of GCaMP6s-positive neurons in the ventral diencephalon, while magenta and green boxes indicate restricted regions of interest for calcium imaging of identified DDN/PTac neurons shown in panels ***C*** and ***D***. Left (L) and right (R) cell groups are indicated in panels ***A*** and ***B***. Identified cell groups: PTar, posterior tubercular anterior rostral; Hdm, hypothalamic dorsomedial; PTac, posterior tubercular anterior caudal; PTp, posterior tuberculum posterior; PTN, posterior tubercular nucleus. ***C***, ***D***, Pseudocolored GCaMP6s fluorescence panels correspond to the time points (indicated by arrows below each Δ*F*/*F* traces (***E*** and ***F***, respectively)). Color indicates fluorescence intensity. ***E***, ***F***, Δ*F*/*F* traces for DDN/PTac neuron groups (L and R) recorded from an individual larva at both 3 dpf (***E***) and 5 dpf (***F***). ***G***, Plot of the mean number of calcium signal peaks detected in immature (3 dpf) and mature (5 dpf) larvae. Gray lines indicate within-subjects repeated measures, while black circles represent the mean (SD) (*n* = 8). ***H***, Plot of the mean magnitude of calcium signal peaks in in immature (3 dpf) and mature (5 dpf) larvae. Gray lines indicate within-subjects repeated measures, while black circles represent the mean (SD) (*n* = 8). ***I***, Plot of the mean total power of calcium signals in immature (3 dpf) and mature (5 dpf) larvae. Gray lines indicate within-subjects repeated measures, while black circles represent the mean (SD) (*n* = 8). Asterisks indicate significant differences at **p < 0.01 and ***p < 0.001.

To compare neural activity between immature and mature larvae, we measured in vivo calcium transients in bilateral DDN/PTac neurons within individual larvae (repeated measures) before (3 dpf) and after (5 dpf) the developmental transformation of locomotor activity. Since neural activity between the bilateral groups of DDN/PTac neurons were highly correlated at both 3 and 5 dpf (Fig. 8*E*,*F*), measures of calcium signals (number, peak, magnitude) were averaged within each preparation. While bilateral correlation was high at both developmental stages, it was significantly greater in mature larvae (Table 6). The number of calcium transient peaks were greater in mature larvae compared with immature larvae (Fig. 8*G*, Table 6). The magnitude of calcium transients was significantly greater in mature larvae compared with immature larvae (Fig. 8*H*, Table 6). Finally, power measures of the calcium transients were significantly greater in mature larvae compared with immature larvae (Fig. 8*I*, Table 6). In a subset of experiments, simultaneous peripheral nerve and calcium imaging recordings revealed that calcium transients were correlated to locomotor activity in both 3 dpf (*n* = 5) and 5 dpf (*n* = 5) larvae (data not shown), consistent with prior studies (Jay et al., 2015; Reinig et al., 2017). These results demonstrated that DDN/PTac activity was greater in mature larvae, which supports the hypothesis that increased dopaminergic signaling through DDN/PTac activity correlates with the transformation of locomotor activity during development.

**Table 6.** Within-subjects comparisons of calcium signal measures during spontaneous locomotor activity between immature and mature larvae

		Developmental stage	
		3 dpf	5 dpf
Pearson's correlation (*r*)	Mean (SD), *n*	0.556 (0.269), 8	0.886 (0.0405), 8
	Mann–Whitney *U*	0.000	
	*p*	<0.001	
Peaks, count (*n*)	Mean (SD), *n*	12.6 (10.6), 8	39.5 (7.95), 8
	*t*	−5.73	
	df	14	
	*p*	5.25 × 10^−5^	
Peaks, magnitude (%)	Mean (SD), *n*	59.6 (16.2), 8	94.6 (23.6), 8
	*t*	−3.49	
	df	14	
	*p*	0.00362	
Signal power (Δ*F*/*F*^2^)	Mean (SD), *n*	0.135 (0.0413), 8	0.321 (0.0938), 8
	*t*	5.14	
	df	14	
	*p*	1.49 × 10^−4^	

Values reported as mean (SD).

## Discussion

The goal of this study was to characterize the functional and molecular mechanisms that correlate to the behaviorally relevant refinement of locomotor activity during development in a vertebrate model system, larval zebrafish. We hypothesized that the coarse locomotor activity produced by immature larvae was due, in part, to a lack of functional D4Rs in locomotor-related spinal neurons, a lack of activity in the DDNs, a lack of endogenous DA release from the DDNs in the spinal cord, or a combination of these. The work presented here demonstrates that the developmental transformation of locomotor activity correlates with an increase in gene expression of all D2-likeR subtypes, an increase in global dopamine, and an increase in activity state of a group of DDNs that project to the spinal cord.

### Spinal locomotor output is less functionally organized in immature larvae

Although spontaneous locomotion produced by immature larvae is less frequent and of longer duration than those produced by mature larvae (Lambert et al., 2012), the functional differences of the spinal locomotor circuit before and after this transformation of locomotor activity have not been characterized. We demonstrated here that properties of NMDA-induced locomotor activity changed during larval development in zebrafish. First, the proportion of immature larvae that produced episodically organized locomotor activity was significantly lower than the proportion of mature larvae. Second, although a low proportion of immature larvae produced organized locomotor activity, the EO scores were significantly lower compared with mature larvae. Third, the episode durations produced by immature and mature larvae were not significantly different. This result is consistent with our previous result that demonstrated NMDA-induced long duration episodes were produced in both spinalized immature and mature larvae (Lambert et al., 2012). Finally, the number of bursts generated by immature larvae were significantly greater compared with mature larvae. Together, these results indicate that the locomotor output produced by immature larvae is functionally less organized.

Locomotor circuit development in zebrafish occurs not only in larval development but also later during development. The El Manira lab demonstrated that V2a interneurons transition from reciprocal excitatory connections at the larval stage to a unidirectional connection pattern in adults (Pallucchi et al., 2022). These V2a interneurons also establish new connections to allow for excitation of all peripheral motor neurons preparing the circuit for escape behaviors (Pallucchi et al., 2022). Such developmental transitions are not unique to zebrafish. In developing mice, the emergence of mature ambulation occurs, in part, through changes in intrinsic properties of commissural interneurons, such as increased spontaneous activity, more narrow action potentials, decreased membrane capacitance, and qualitative changes in the firing properties in response to serotonin (Abbinanti et al., 2012).

The functional differences observed between immature and mature larvae could be due to a lower excitability state of the spinal locomotor network at the immature stage. In support of this, dopamine application was previously shown to increase the excitability state of the locomotor network in mice, in part, by decreasing the afterhyperpolarization of spinal motor neurons and increasing glutamatergic transmission onto spinal motor neurons (Han et al., 2007; Han and Whelan, 2009). Additional work in neonatal mice demonstrated that increased network excitability increased the power of the fast locomotor rhythm and decreased the level of dopamine required to evoke locomotor rhythms (Sharples and Whelan, 2017). Consistent with these outcomes, our results indicate that the lower excitability state of immature larvae (e.g., low proportion of episodically organized NMDA-induced fictive locomotion) could be due to lower levels of dopamine, fewer functional D2-likeRs present in locomotor-related neurons, or both. While we did not measure the levels of D1-likeRs in this study, it is possible that an increase in D1-likeRs present in locomotor-related neurons (Shontz et al., 2018) could also contribute to the increase in excitability state of the network (Han and Whelan, 2009; Sharples et al., 2020). Future studies are needed to assess D1-likeR contributions to the excitability state or locomotor pattern.

The increase in episodic organization during development could also be mediated by nondopaminergic mechanisms. A recent study in the neonatal mouse demonstrated that both the Na^+^/K^+^ ATPase pump current and the hyperpolarization-activated cation current acted to organize the episodic structure of the circuit output (Sharples et al., 2022). Specifically, a reduction of the hyperpolarization-activated cation current decreased episode duration and increased interepisode intervals, while reduction or enhancement of the Na^+^/K^+^ ATPase pump current led to continuous bursting or increased interepisode interval, respectively (Sharples et al., 2022).

### D4Rs are functional in immature larvae

Previously, we demonstrated that dopaminergic signaling through inhibitory dopamine receptors was necessary to maintain the mature locomotor phenotype following the developmental transformation of locomotor activity (Lambert et al., 2012). Therefore, we reasoned that the coarse locomotor activity (swimming) produced by immature larvae may be due to a lack of functional D4Rs in locomotor-related spinal neurons.

First, we confirmed that NMDA-induced episodically organized locomotor activity was sufficiently stable in immature larvae for the duration of the experimental paradigm to permit measurement of potential effects on the locomotor pattern in response to application of D2-likeR agonists. None of the measures of episodic or bursting activity were affected by the duration of NDMA application, thus confirming that NMDA-induced episodically organized locomotor activity produced by immature larvae was sufficiently stable over the course of the experiment to assess the effects of pharmacological perturbation of dopamine receptors. Further, since DMSO was used as a vehicle for the D4R agonist, we tested for potential effects of vehicle administration on NMDA-induced locomotor activity. Application of the vehicle did not produce significant differences in episodic or bursting activity. However, episode organization trends down without reaching significance. The low success rate for generating episodically organized locomotor activity led to lower sample sizes for this group, which reduced the statistical power for this measure, so the interpretation of the effect of D4R agonist application on episode organization is uncertain due to confounding effects of DMSO.

Next, we tested the hypothesis that inhibitory dopamine receptors were functional in immature larvae. Application of a broad D2-likeR agonist produced a significant decrease in EO scores during NMDA-induced fictive swimming in immature larvae. While there was a trend toward a decrease in both episode duration and mean bursts per episode, the effect did not reach statistical significance. Likely, the statistical power was insufficient since the sample size (*n* = 5) was small for this group, which was reflected by the low success rate (15%; 35 out of 236 experiments) for generating NMDA-induced episodically organized locomotor activity in immature larvae. Importantly, the EO scores significantly decreased.

Finally, application of a specific D4R agonist significantly reduced episode duration, mean number of bursts per episode, EO score, and mean burst frequency during NMDA-induced fictive swimming in immature larvae. We are cautious in our interpretation of the effects of the D4R agonist on EO score since this also decreased, without reaching significance, in the DMSO control group.

Overall, these results demonstrate that pharmacological application of D2-likeR or D4R agonists to immature larvae does not reduce episode duration. Rather, it functionally disrupts the organization of bursts into episodes but does not, overall, inhibit NMDA-induced locomotor activity. Unfortunately, it is not possible to disambiguate D2R and D3R subtype function in immature larvae because of the high affinity of the D2-like agonist (quinpirole) to both receptor subtypes (Seeman and Van Tol, 1994).

### Gene expression of all D2-likeRs is greater in mature larvae

Our previous work reported that the developmental transformation of locomotor activity was dependent on D2-likeR and D4R signaling (Lambert et al., 2012). In addition, we demonstrated in this study that D4Rs were functional in immature larvae. Therefore, we tested the hypothesis that developmental changes in gene expression levels of all D2-likeR subtypes in the spinal cord correlate with the developmental transformation of locomotor activity. The qRT-PCR analysis confirmed that all D2-like receptor subtype transcripts were present in immature larvae (Boehmler et al., 2004, 2007), and all, except *drd2l*, were significantly greater in mature larvae. The relative increase in gene expression of these receptor genes correlates with the developmental transformation of locomotor activity, which supports our hypothesis that an increase in D2-like receptor subtypes in mature larvae is, at least, one mechanism that contributes to the developmental transformation of locomotor activity.

A caveat to the interpretation of the qRT-PCR experiments is that the D2-like receptor transcripts were measured from the bodies of zebrafish larvae, which included skin, muscle, and notochord, but excluded the head, yolk sac, yolk finger, and gut. Although these transcripts were not measured from isolated spinal cords, we are confident that the measures reflected changes in transcript expression in spinal tissue. First, prior studies using whole-mount in situ hybridization demonstrated that all D2-like receptor transcripts [*drd2a*, *drd2b* (current nomenclature: *drd2l*), *drd2c* (current nomenclature: *drd2b*), *drd3*, *drd4a*, *drd4b* (current nomenclature: *drd4rs*), *drd4c* (current nomenclature: *drd4b*)] were present in zebrafish larvae at both 2 and 5 dpf (Boehmler et al., 2004, 2007). Second, the spatial expression of these transcripts revealed that each was present in the brain, spinal cord, or both and only three (*drd3*, *drd2c*, *drd4c*) were present outside of the central nervous system (Boehmler et al., 2004, 2007). Specifically, the *drd4c* gene was present in the endoderm at 24 hpf, the *drd2c* gene was present in the notochord at 24 hpf, and the *drd3* gene was present in the myotomes at 24 hpf. None of the three transcripts were noted at later developmental timepoints however, as only expression in the head or rostral spinal cord was shown for the 5 dpf developmental stage (Boehmler et al., 2004, 2007). Ultimately, we recognize the possibility that changes in transcript expression may be due to changes in both spinal cord tissue and other tissues (skin, muscle, notochord); however, the spatiotemporal expression of these transcripts was mostly restricted to retina, brain, and spinal cord and strongly indicates that by removing the head, we primarily assessed spinal cord transcript levels. A future study could repeat these transcript measures from isolated spinal cords (Kelly et al., 2023). Finally, although we did not directly measure protein levels for D2-like receptor subtypes, our functional data indicate that these receptors are functional in immature larvae.

Additionally, characterization of the spatial distribution of D2-like receptor subtypes on identified locomotor-related spinal neurons would provide insights into the functional mechanisms responsible for the developmental transformation of locomotor activity. For example, one study demonstrated that V3 spinal interneurons were hyperpolarized in response to D2-likeR agonist (quinpirole) application in mice (Sharples et al., 2020), while a second study showed that DDN projections (DDT) likely synapse on spinal motor neurons and that these synapses colocalized with D4Rs in larval zebrafish (Son et al., 2020). Further, dopamine application increased motor neuron excitability and was necessary, but was not sufficient, to generate oscillatory rhythm in Hb9 interneurons in neonatal mice (Han et al., 2007). These studies suggest dopamine plays a role in both increasing the excitability state of the network and sculping the locomotor pattern at the mature stage. Therefore, in future experiments, we plan to identify locomotor-related spinal neurons that express D2-like receptor subtypes in both immature and mature larvae by mapping their locations and relative abundance using third-generation in situ hybridization chain reaction.

### More dopamine is present in mature larvae

We demonstrated that the D4R subtype is functional in the spinal locomotor circuits of immature larvae and that gene expression of all D2-like receptor subtypes are increased in the bodies of larval zebrafish across the developmental transformation of locomotor activity. Next, we tested the hypothesis that an increase in global DA levels correlated with the transformation of locomotor activity. Whole-body (global) DA levels increased significantly during the developmental transformation. Although our data do not directly demonstrate that more DA was present in nervous tissue of mature larvae, it provided evidence that more DA was present globally in mature larvae. This result is consistent with increased endogenous dopamine levels in the spinal cord during development from the neonatal stage to adult in mice (Sharples et al., 2020). Interestingly, previous studies showed that low levels of dopamine preferentially activated high affinity D2-likeR subtypes (Clemens and Hochman, 2004; Dreyer et al., 2010; Clemens et al., 2012; Jay et al., 2015; Sharples et al., 2020), while high levels of dopamine, in addition, activated low affinity D1-like receptors (Barrière et al., 2004; Dreyer et al., 2010; Clemens et al., 2012; Jay et al., 2015; Sharples et al., 2020) or coactivated D1-like and D2-like receptors (Zhu et al., 2007; Sharples et al., 2020). Thus, the global increase in dopamine levels measured between immature and mature zebrafish larvae could increase modulatory tone in the spinal cord (Jay et al., 2015), increase network excitability state through D1-like receptors (Han et al., 2007; Han and Whelan, 2009), and/or shorten episode durations mediated by inhibitory D2-like receptors (Thirumalai and Cline, 2008; Lambert et al., 2012). Since the global dopamine measured was an indirect assessment of dopamine released in the spinal cord, it will be imperative to utilize a direct approach to determine the differences in dopamine levels released in immature and mature larvae. For example, expression of a dopamine sensor (GRAB_DA_ or dLight) under a pan-neuronal promoter would permit the measure of dopamine levels using an in vivo optical paradigm (Patriarchi et al., 2018; Sun et al., 2018). Alternatively, amperometry potentially could be used to measure dopamine release in the spinal cord (Shang et al., 2015; Dumitrescu et al., 2022).

### DDNs are more active in mature larvae

We reasoned that since a greater amount of dopamine was present in mature larvae, then an increase in activity in DDN/PTac neurons in mature larvae would increase the yield of dopamine release on spinal locomotor circuits. Thus, we used DDN/PTac activity as a proxy for dopamine release to test the hypothesis that increased DDN/PTac activity from the immature to the mature stage of larval development correlated with the locomotor transformation.

Although the DDN/PTac neurons generated a range of activities in both immature and mature larvae, the measured calcium transients increased within all preparations during development. Specifically, the number and magnitude of calcium transient peaks and the power measures of calcium transients were significantly greater in mature larvae compared with immature larvae. Additionally, while the bilateral PTac neurons were highly correlated in both immature and mature larvae, the correlation was significantly higher in mature larvae. These results support the hypothesis that increased dopaminergic signaling through DDN/PTac activity correlates with the transformation of locomotor activity during development. Importantly, ∼50% of the DDN/PTac neuron population projects to the spinal cord (Tay et al., 2011), so the paradigm used here to monitor activity in DDN/PTac neurons included, but was not limited to, spinally projecting neurons. Regardless, our data demonstrates that activity in at least a subset of the spinally projecting DDN/PTac neurons increased between immature and mature larvae.

Calcium imaging experiments were performed in intact larval preparations due to the location of the DDN/PTac neurons the head (ventral diencephalon) and the within-subject design of the experiment (repeated measures at 3 and 5 dpf). Since activity in the DDNs has been correlated with locomotion in mature larvae (4–6 dpf; Jay et al., 2015; Reinig et al., 2017), the difference in DDN/PTac neural activity between 3 and 5 dpf larvae could be a consequence of increased locomotor activity driven by higher-order centers in mature larvae. Previous work from Thirumalai and Cline demonstrated that dopaminergic signaling suppressed locomotion through supraspinal targets in 3 dpf larvae (Thirumalai and Cline, 2008). It has also been hypothesized that dopaminergic signaling from posterior tubercular nucleus projections to the hindbrain may increase locomotor output (Reinig et al., 2017; Jha and Thirumalai, 2020). Whether an increase in locomotor activity drives an increase in DDN/PTac neural activity or vice versa, we predict that greater DDN/PTac activity in mature larvae likely corresponds to greater release of DA on spinal locomotor circuits since greater levels of DA are present.

In conclusion, this work provides convergent correlative evidence for multiple mechanisms underlying the developmental transformation of locomotor activity in vertebrates. We showed that the spinal locomotor output is more functionally organized after the developmental transformation, and that D4Rs were functional in the spinal locomotor circuits of, at least, a subset of immature larvae. Importantly, gene expression of all D2-like receptor subtypes except *drd2l*, levels of dopamine, and activity of diencephalic dopaminergic neurons were significantly greater in mature larvae compared with immature larvae. Overall, the integration of these results indicates an important developmental role for dopaminergic signaling in the refinement of locomotor activity in vertebrates.

## Synthesis

Reviewing Editor: David Schoppik, New York University Grossman School of Medicine Decisions are customarily a result of the Reviewing Editor and the peer reviewers coming together and discussing their recommendations until a consensus is reached. When revisions are invited, a fact-based synthesis statement explaining their decision and outlining what is needed to prepare a revision will be listed below. The following reviewer(s) agreed to reveal their identity: Simon Sharples, Adam Douglass. Note: If this manuscript was transferred from JNeurosci and a decision was made to accept the manuscript without peer review, a synthesis may not be available.

Major Comments

Inference from Whole-Body Measurements

D2-like receptor expression was assessed using whole-body qRT-PCR, which includes but is not limited to the spinal cord. Similarly, dopamine levels were measured using mass spectrometry on intact larvae. In both cases, changes within spinal circuits are inferred rather than directly measured. It is not clear why isolated spinal cords were not analyzed, particularly given the central role of spinal circuits in the authors' interpretations. These limitations should be more explicitly acknowledged, and the corresponding conclusions tempered accordingly. This limitation should be emphasized in the Discussion.

Terminology Related to Circuit Organization

Throughout the manuscript, the authors frequently refer to changes in "locomotor circuit organization." However, the primary measurements in this study reflect locomotor output, particularly features of episodic activity, rather than direct assessments of circuit architecture or synaptic connectivity. This distinction is especially important in the context of neuromodulation, where fully mature and functional circuits can nonetheless exhibit markedly different outputs depending on neuromodulatory state. For example, in the adult crustacean stomatogastric ganglion, neuromodulators can profoundly reshape network output without altering underlying circuit structure. The manuscript would benefit from revised language that more precisely reflects what is being measured-namely, changes in locomotor output and episodic structure-rather than implying direct changes in circuit organization. This issue should be addressed consistently throughout the text.

Statistical Reporting

Statistical details are not consistently reported in the text or figures. All relevant statistics (including test statistics, degrees of freedom, p values, and sample sizes) should be provided. If the authors intend to consolidate statistical results in summary tables for readability, this should be clearly stated in both the Methods and Results, with appropriate direction to the relevant tables. Of note, variance should be represented graphically by including the CI or similar metric for the matched sample comparisons in Figs 2-4.

Focus on Episodic Activity

The locomotor analyses are largely restricted to features of locomotor episodes. This focus is a strength of the study and distinguishes it from many prior investigations of developing locomotor circuits, which often emphasize rhythm frequency or burst timing. Only a limited number of studies have begun to examine the episodic structure of locomotion, and this novelty should be highlighted more clearly. At the same time, it would be informative to ask whether developmental changes occur in burst frequency or pattern within episodes, as such changes could reflect differential maturation of rhythm-generating versus episode-generating components of the locomotor CPG.

DMSO Control Effects

A major concern is that the DMSO control data exhibit trends similar to those observed with D2 and D4 agonists. Although these effects do not reach statistical significance, they are consistently trending (Table 3), and sample sizes are small, likely limiting statistical power. This raises concerns about interpretation of the pharmacological effects. The authors should address this issue more directly, either by increasing sample sizes, providing power analyses, or more cautiously interpreting these results.

Importance of reframing the work with respect to prior knowledge

The authors hypothesize that the delayed appearance of mature kinematics could arise from an absence of functional D2-family receptors at early developmental stages, a possibility they explore using qRT-PCR and pharmacological manipulation of fictive motor activity. While the observation of increased receptor expression at 5 dpf is new, most of the D2R-family members were already shown to be present in the embryonic spinal cord through ISH (Boehmler et al. 2004; Reimer et al. 2013). The authors themselves point this out, but only after setting up and describing the experiments of Figs 2-4 with a bit of a straw-man hypothesis (lines 346-8). They also do not mention that pharmacological manipulations in their own prior work (Lambert et al. 2012, Fig 2) already strongly suggested that the receptors are functional during early development. The data included here are still new because they examine fictive behavior during acute drug exposure and not the developmental effects of such manipulations, but the manuscript needs to be much clearer about what was already known, and what we actually learn from these experiments.

Importance of acknowledging the paper's limitations

1. Most of the paper's central findings are correlative. This includes the increases in receptor expression, the increase in A11 activity, and the increase in tissue DA levels following locomotor maturation. While such correlations are useful, the lack of causal manipulations in this work prevents one from drawing strong mechanistic conclusions. These limitations should be communicated more clearly throughout, e.g. by replacing statements like "this work provides convergent evidence for multiple mechanisms underlying the developmental transformation of locomotor activity in vertebrates." (lines 567-8), which imply more specific insight than is actually gained.

2. Higher levels of DA in tissue extracts don't necessarily mean that more DA is released following maturation, as the extraction procedure recovers both intra- and extracellular pools. Similarly, because the coupling between spiking and dense-core vesicle fusion can be complex or even nonexistent, the finding that A11 neurons are more active in older fish does not mean that spinal DA release is elevated proportionately. It's important to note that while these measurements are consistent with increased D2 receptor signaling at 5 dpf, they do not demonstrate it directly.

3. The identification of spinal DA afferents in the context of calcium imaging (Fig 8) is ambiguous. While the ROI indicated in this figure includes the DC2/4 subset of PTN, it also seems to contain neurons/neuropil from adjacent th1+ nuclei. Additionally, because roughly 50% of the DC2/4 groups do not project to the spinal cord (Tay et al. 2011), these signals cannot be unambiguously linked to the neurons of interest. Both of these problems are compounded by the use of wide-field illumination and an analytical procedure that measures fluorescence intensity within a large ROI instead of segmenting single neurons.

Finally, while the attempt to measure the stability of their fictive prep over time is commendable, the rationale for defining "early" and "late" as t = 30 and 40 min, respectively within a 60 min recording is unclear. Why just a 10 minute gap, and why in the middle of the recording period?

Minor Comments

Title - The title is relatively vague and does not clearly convey the novelty of the study or distinguish it from the authors' previous work. A more specific title would strengthen the manuscript.

Line 100 - Consider citing recent work from the Mentis lab on descending dopaminergic and noradrenergic contributions to dysfunction in spinal muscular atrophy (Cell Reports, 2024).

Line 325 - It is unclear whether this analysis was performed on spontaneously occurring episodes or in the presence of NMDA, as described in the subsequent paragraph. This should be clarified.

Line 337 - The text again refers to "organization of the locomotor circuit." It would be more accurate to specify that the observed changes likely pertain to mechanisms responsible for generating or structuring episodes of activity. Intra-episode activity appears relatively well organized even at earlier stages, aside from modest frequency differences. This distinction deserves clearer emphasis.

Line 351 - Please clarify what is meant by "continuous" NMDA application. Is this contrasted with pulsed or transient application?

Line 359 - A non-significant change should not be described as a decrease. The text should be rephrased to indicate that quinpirole did not significantly alter episode duration. While the inclusion of individual preparation data is appreciated, the high variability-largely driven by a single preparation-likely reduces statistical power, especially given the small sample size. Rephrasing the conclusions to more closely reflect the statistics would strengthen this section. Given that experiments with the D4 agonist included larger sample sizes, increasing sample sizes here and providing power calculations would more strongly support the conclusions. Although this issue is acknowledged in the Discussion, additional attempts to address it experimentally would be valuable.

Figures

Figures 6 and 7 appear to be reversed in the PDF.

Calcium Imaging Experiments- Work from Jay, et al., has demonstrated that DDT neuron firing is tonic at rest and bursting during locomotor activity, indicating state dependence. Was locomotor state monitored during calcium imaging? Were there discernible differences in the temporal organization or patterns of calcium transients between immature and mature stages? Even a qualitative description of these signals would add interpretive value.

The observation that "episode durations generated by immature larvae were not significantly different (Table 2) compared to mature larvae" seemingly contradicts the findings of both Lambert et al (2012) and Thirumalai and Cline (2014), and needs comment.

Discussion

The Discussion is relatively brief and largely reiterates the main findings, with limited engagement with the broader literature. It would benefit from additional references and a deeper integration of these results with prior work on dopamine's role in developing spinal circuits, including studies from the Sillar, Miles, Whelan, Clemens, and Thirumalai laboratories.

The Discussion would also be strengthened by proposing potential mechanisms underlying the developmental increase in episodic organization beyond dopamine receptor expression alone. Dopamine receptors act on a range of ion channels and pumps that have been implicated in locomotor and episodic control within the spinal cord. Relevant examples can be found in work from the Sillar, Whelan, Cymbalyuk, and Miles laboratories.

The authors should highlight relevant studies from the El Manira Lab and place their findings in the broader context of how swim circuits mature from larval to adult stages. This literature provides important perspective on developmental changes in locomotor circuits and would strengthen the framing of the current results.

The authors should also highlight Vatsala Thirumalai's publications on locomotor maturation through DA, which provides the opportunity to discuss potential supraspinal mechanisms, which need not be done is great detail but would help anchor this work in its larger context.

Finally, although this manuscript focuses primarily on D2-like receptors, the Discussion would benefit from integrating potential roles for D1 receptors. Even if D1 receptors are not driving the observed developmental changes in locomotor output, they may still modulate circuit function-particularly given the developmental increases in dopamine levels and DDT neuron activity, which could engage lower-affinity D1 receptors. Placing these possibilities in the context of existing studies on dopaminergic modulation of spinal circuits would provide a more comprehensive framework for the findings.
